# Hybrid Materials Based on Silica Matrices Impregnated with Pt-Porphyrin or PtNPs Destined for CO_2_ Gas Detection or for Wastewaters Color Removal

**DOI:** 10.3390/ijms21124262

**Published:** 2020-06-15

**Authors:** Diana Anghel, Anca Lascu, Camelia Epuran, Ion Fratilescu, Catalin Ianasi, Mihaela Birdeanu, Eugenia Fagadar-Cosma

**Affiliations:** 1 Institute of Chemistry “Coriolan Dragulescu” of Romanian Academy, M. Viteazul Ave, No. 24, 300223 Timisoara, Romania; danghel@acad-icht.tm.edu.ro (D.A.); ecamelia@acad-icht.tm.edu.ro (C.E.); ionfratilescu@acad-icht.tm.edu.ro (I.F.); ianasic@acad-icht.tm.edu.ro (C.I.); 2 National Institute for Research and Development in Electrochemistry and Condensed Matter, P. Andronescu Street 1, 300224 Timisoara, Romania; mihaelabirdeanu@gmail.com

**Keywords:** Pt-pophyrin, PtNPs, silica hybrid materials, wet carbon dioxide adsorption, toxic dyes

## Abstract

Multifunctional hybrid materials with applications in gas sensing or dye removal from wastewaters were obtained by incorporation into silica matrices of either Pt(II)-5,10,15,20-tetra-(4-allyloxy-phenyl)-porphyrin (PtTAOPP) or platinum nanoparticles (PtNPs) alone or accompanied by 5,10,15,20-tetra-(4-allyloxy-phenyl)-porphyrin (TAOPP). The tetraethylorthosilicate (TEOS)-based silica matrices were obtained by using the sol-gel method performed in two step acid-base catalysis. Optical, structural and morphological properties of the hybrid materials were determined and compared by UV-vis, fluorescence and FT-IR spectroscopy techniques, by atomic force microscopy (AFM) and high resolution transmission electron microscopy (HRTEM) and by Brunauer–Emmett–Teller (BET) analysis. PtTAOPP-silica hybrid was the most efficient material both for CO_2_ adsorption (0.025 mol/g) and for methylene blue adsorption (7.26 mg/g) from wastewaters. These results were expected due to both the ink-bottle mesopores having large necks that exist in this hybrid material and to the presence of the porphyrin moiety that facilitates chemical interactions with either CO_2_ gas or the dye molecule. Kinetic studies concerning the mechanism of dye adsorption demonstrated a second order kinetic model, thus it might be attributed to both physical and chemical processes.

## 1. Introduction

The remarkable interest in porphyrin-functionalized silica hybrid materials is justified by their main characteristics, such as biocompatibility, high chemical resistance and most of all their rich opto-electronical properties [1]. In addition, due to their high specific surface area (usually over 300 m^2^/g) [2], tailored pore shapes and sizes, these hybrid porphyrin-silica materials have tremendous applications in detection (ascorbic acid) [3], the removal of toxic heavy metals [4] and catalysis [5,6,7]. The multiple reuse and easy separation are the major benefits brought by these materials.

Over time, a large interest has been shown for using these porphyrin-functionalized silica hybrid materials for the detection of CO_2_ [8], oxygen [9,10,11], hydrochloric acid [12] or for organic volatile substances (trinitrotoluene vapors [13] and hydrocarbon solvents [14]). 

The presence of porphyrin units on the pore surface of silica matrices can favor the trapping of small molecules such as CO_2_, by forming new physical or even chemical bonds [15]. For example, mesoporous silica impregnated with various amounts of polyethyleneimine demonstrated CO_2_ adsorption capacities from 1.65 [16] to 2.045 mmol/g [17]. Tetraethylorthosilicate (TEOS)-based silica gel impregnated with tetratolylporphyrin can detect CO_2_ in a range of concentrations from 1.21 to 4.45 mM [18].

The removal of toxic dyes, such as acid yellow (adsorption capacity of 9.7 mg/g) [19], rhodamine B (adsorption capacity in the range 25–41 mg/g) [20], Congo red (adsorption capacity of 66.5 mg/g) [21] and methylene blue (MB), from wastewaters was attempted using silica-based materials as adsorbents. In this context, mesoporous silica impregnated with dodecyl-trimethyl-ammonium bromide deposited on a titania honeycomb structure can adsorb 95.95 mg MB/g [22]. Another TEOS -based mesoporous silica material functionalized with a symmetric triblock copolymer comprising poly(ethylene oxide) and poly(propylene oxide) has the capacity to adsorb 49.25 mg MB/g [23]. 

Decomposing dyes under the influence of light, catalyzed by porphyrin-hybrid materials, represents a different method to purify wastewaters. For example, Cu(II)- 5,10,15,20-tetrakis(4-(hydroxyl)phenyl) porphyrin assembled into a magnetic photocatalyst (magnetite- silica hybrid) was able to degrade MB under blue LED light, (λ = 465 nm) with 98% efficiency [24]. Another metalloporphyrin, Cu(II)-*meso*-tetra-(4-carboxyphenyl)- porphyrin [25], acts as photocatalyst for the degradation of MB under visible light irradiation (yield of 59.3%).

There are only a few mentions in the literature concerning the capacity of porphyrin-hybrid materials to adsorb dyes. Recent work focused on the obtaining of a porous organic polymer, generated from 5,10,15,20-tetra-(1,1′-biphenyl) porphyrin, capable to adsorb 17.9 mg MB/g [26], but their anterior results using alkaline metal organic frameworks containing Ca(II)-5,15-di(4-carboxyphenyl)-porphyrin have the best ever reported performance, with an adsorption capacity of 952 mg MB/g [27].

In order to obtain silica matrices with tailored properties, sol-gel immobilization processes both in acid and acid-base catalysis are preferred because these methods offer easy and mild synthetic conditions. As a general rule, porphyrins entrapped in silica gels manifest bathochromic effects of the Q bands at acidic pH and frequently exhibit hyperchromic effects when acid-base catalyzed methods are used, due to both hydrophobic co-facial H-type and to side-by-side J-type aggregation of porphyrins [28].

**Figure 1:**
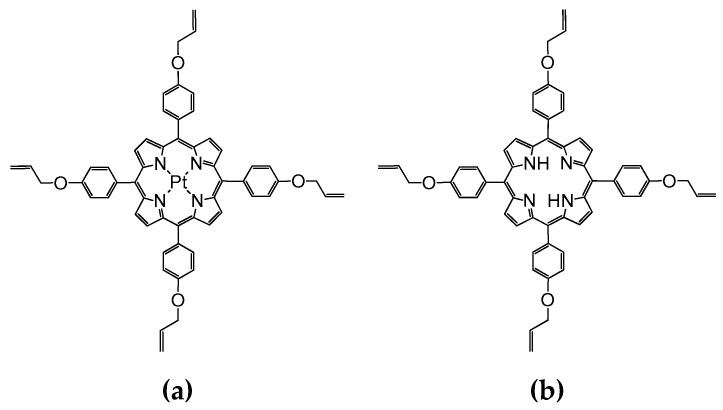
The structures of (**a**) Pt(II)-5,10,15,20-tetra-(4-allyloxy-phenyl)-porphyrin (PtTAOPP) and (**b**) 5,10,15,20-tetra-(4-allyloxy-phenyl)-porphyrin (TAOPP).

Incorporating Pt(II)-5,10,15,20-tetra-(4-allyloxy-phenyl)-porphyrin (PtTAOPP) and platinum nanoparticles (PtNPs) alone or accompanied by 5,10,15,20-tetra-(4-allyloxy-phenyl)-porphyrin (TAOPP) into tetraethylorthosilicate (TEOS)-based silica matrices will allow us to tailor the porosity, the surface area, the aggregating phenomena and the optical features of the novel hybrid materials. Obtaining three types of multifunctional material, presented in Table 1, with improved performances for CO_2_ detection and for the color removal of dyes from wastewaters are the major targets of this work. The structures of PtTAOPP and of TAOPP are presented in Figure 1.

## 2. Results and Discussions

### 2.1. Physical Characterisation of the Obtained Silica Hybrids

#### 2.1.1. UV-Vis Spectroscopy of Sol Samples, during the Synthesis of Hybrid Materials

The methods for obtaining the silica gel control sample and also the hybrid porphyrin-silica materials are presented in detail in Section 3.3. The silica gels were prepared by a sol-gel method, which was conducted via two step acid-base catalysis and the porphyrins and nanoparticles incorporated are presented in Table 1. 

**Figure 2:**
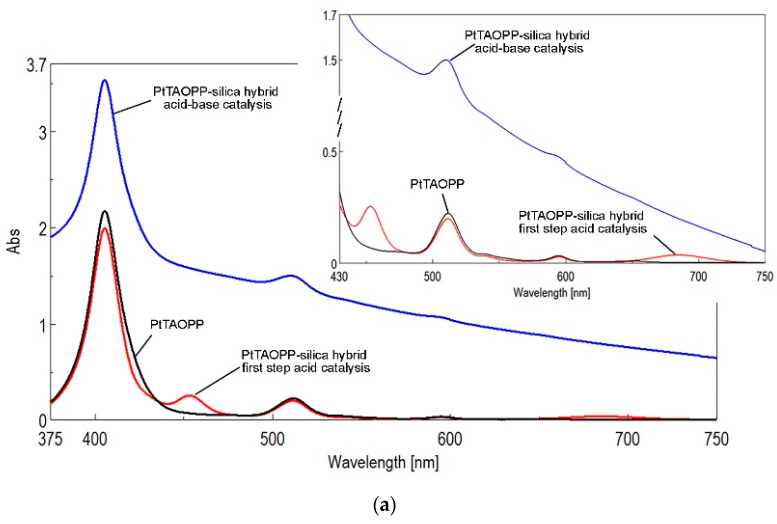

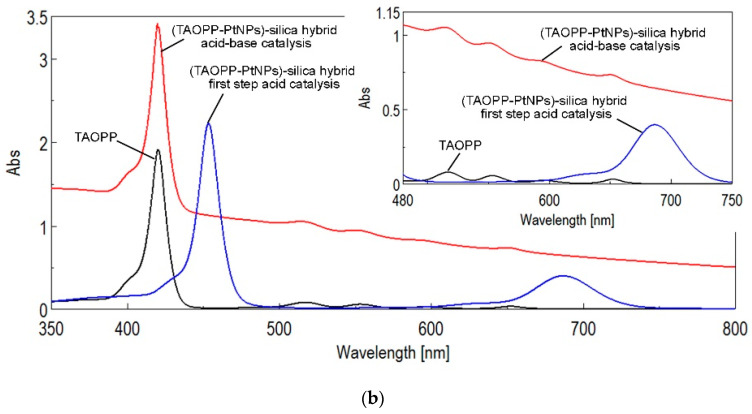
The overlapped UV-vis spectra of (**a**) PtTAOPP and its PtTAOPP-silica hybrid at the end of different types of catalysis; (**b**) TAOPP and its (TAOPP-PtNPs)-silica hybrid at the end of acid-step and acid-base two-steps catalysis, in THF. All samples have the same concentration (c = 4.5 × 10^−6^ M).

The UV-vis spectra of PtTAOPP-silica hybrid, registered both at the end of acid step catalysis and at the end of the second step base catalysis, revealed important changes in comparison with the PtTAOPP spectrum, regarding the number of the main absorption bands, their intensity, position and shape. As easily can be seen in Figure 2a, the Soret band of PtTAOPP, corresponding to a strong transition to the second excited state (S_0_→S_2_), preserved in each case its position at around 406 nm. The Q band, assigned to a weak transition to the first excited state (S_0_→S_1_), located at 510 nm, is also found in both types of catalyzed PtTAOPP-silica hybrid. At the end of acid-step catalysis, two intense new bands appeared at 453 and 684 nm, being assigned to the processes of J-type aggregation of PtTAOPP, in agreement with the literature [29].

The capacity of the porphyrin component to preserve its absorption properties, when impregnated in minute amounts in silica matrices, is amazing and already discussed in our works [3,10,30] Although the PtTAOPP content is 25,000 times lower in comparison with that of TEOS, the intensity of the Soret and its Q bands are identical as in the initial solution. This is the proof of a great optical synergy between porphyrins and silica materials.

The UV-vis spectroscopy of (TAOPP-PtNPs)-silica hybrid is shown in Figure 2b. In this case, the influence of PtNPs incorporated simultaneously with the porphyrin-base TAOPP has no real significance and it behaves in a similar way to the bare porphyrin TAOPP. So, the Soret band, attributed to the π–π* transition from the ground state S_0_ to the second excited singlet state S_2_, is intense and located at 420 nm. The four Q bands, attributed to π–π* transitions from the ground state S_0_ to the different vibration levels of the first excited singlet state S_1_, are present in the spectra around 515 (QIV), 552 (QIII), 595 (QII) and 651 nm (QI), respectively. 

Major differences are registered at the end of acid step catalysis, when the porphyrin base TAOPP suffers changes due to its di-protonation, namely: the Soret band is hyperchromically and bathochromically shifted by 34 nm. In addition, the QIV, QIII and QII bands are not present because the symmetry of the porphyrin structure is increased from D_2h_ to D_4h_ and the QI band is also increased in intensity by ten-fold and red shifted with 36 nm.

#### 2.1.2. UV-Vis Spectroscopy of Solid Samples

**Figure 3:**
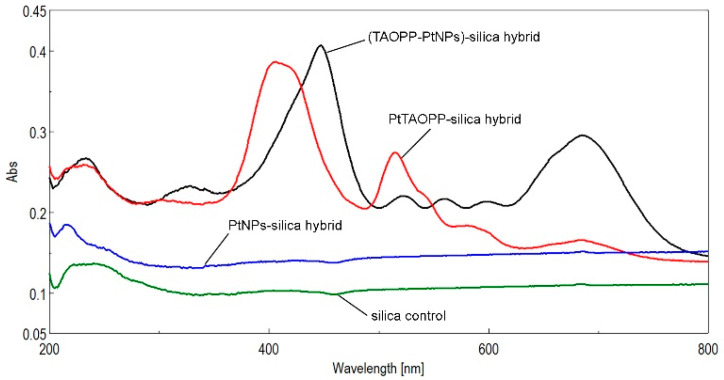
The superposed UV-vis spectra of the four silica materials, as powders: silica control (green line); PtNPs-silica hybrid (blue line); PtTAOPP-silica hybrid (red line) and (TAOPP-PtNPs)-silica hybrid (black line).

As expected, the silica control and the PtNPs-silica hybrid materials have no signal in the UV-vis region from 300 to 800 nm (Figure 3). 

Significant differences can be seen by comparing the two UV-vis spectra of PtTAOPP-silica and (TAOPP-PtNPs)-silica hybrid materials, shown in Figure 3. The Soret band of (TAOPP-PtNPs)-silica hybrid is red shifted by almost 40 nm and manifests a slight hyperchromic effect in comparison with the PtTAOPP-silica hybrid material; its four Q bands are present, with the QI band being wider, red shifted to 700 nm and also increased in intensity. This feature is explained by the extended hydrogen bonding between the oxygen from the allyloxy groups belonging to the porphyrin and the OH from the silica surrounding. It is well established that the Q bands intensity and position in the UV-vis spectra is influenced by the environment [31]. The most important feature in the absorption spectrum of PtTAOPP-silica hybrid material is the hyperchromicity of the Q band around 515 nm.

#### 2.1.3. Fluorescence Spectra of Solid Samples

**Figure 4:**
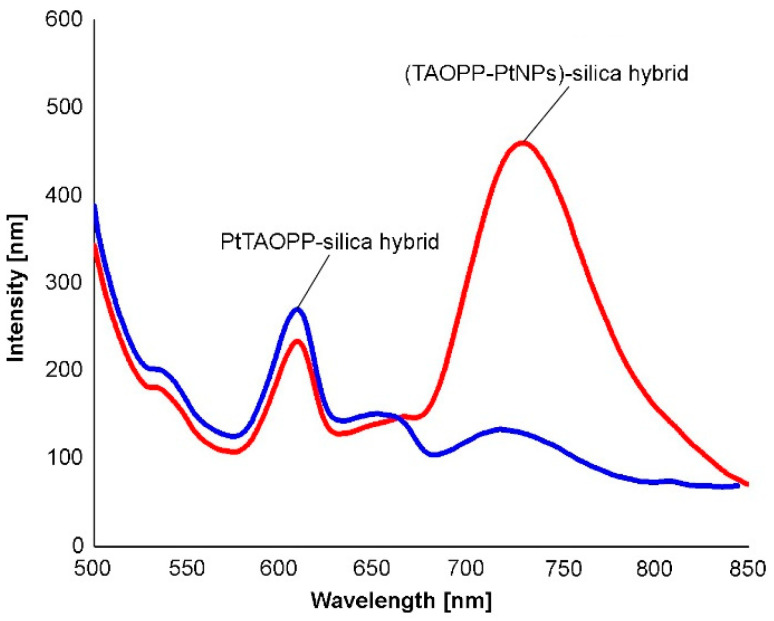
Emission spectra of solid samples of PtTAOPP-silica hybrid and (TAOPP-PtNPs)-silica hybrid.

The common characteristics of PtTAOPP-silica hybrid and (TAOPP-PtNPs)-silica hybrid (Figure 4) is that both exhibit a clear band around 610 nm, assigned to the Q(0,0) transition accompanied by another one in the range of 720–730 nm, representing the Q(0,1) transition. The Q(0,1) band is stronger in the case of (TAOPP-PtNPs)-silica hybrid and weaker in case of PtTAOPP-silica hybrid. Due to emission at higher wavelengths λ > 630 nm, these materials are promising photosensitizers for photodynamic therapy of cancer (PDT) [1], a non-invasive medicine procedure. In addition, positive Stokes shifts (the difference between wavelength values of the Q(0,0) emission band and wavelength of Soret absorption band) around 200 nm are obtained, thus this material makes a clear distinction of the emission from scattered light [32]. Because of rapid vibronic relaxation from S_2_ to S_1_, only the emissions at 610 nm and 720–730 nm were observed and there were no emission peaks near the Soret band. A pertinent explanation is that the strong π–π interaction in porphyrins leads to major aggregation and subsequently to fluorescence quenching and emission red shift [33].

#### 2.1.4. The FT-IR Spectroscopy

**Figure 5:**
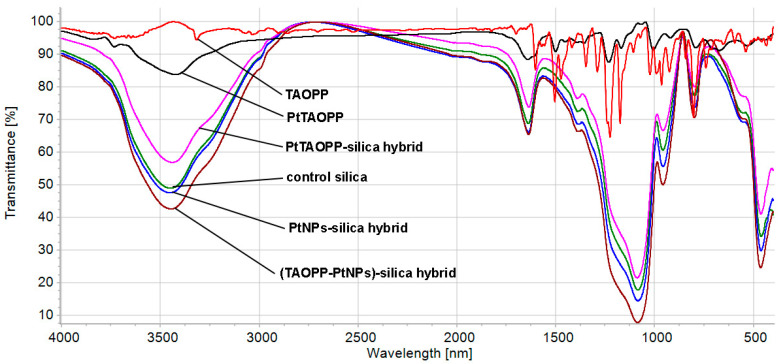
The FT-IR overlapped spectra of the TAOPP, PtTAOPP and of the four silica materials, as powders in KBr pellet.

The overlapped FT-IR spectra of all involved compounds and hybrid materials are presented in Figure 5.

The FT-IR spectrum of the free TAOPP porphyrin has as characteristic feature in the weak band located at 3410 cm^−1^ assigned to the internal core N-H bond, sharp and intense bands at 1100 and 1200 cm^−1^ due to vibrations of the four C-O-C bonds, aromatic sharp bands at 1500–1600 cm^−1^ and C-H and N-H wagging and twisting modes vibrations from pyrrole between 700 and 950 cm^−1^ [34].

The FT-IR spectrum of PtTAOPP does not have the band located at 3410 cm^−1^, certifying that the Pt metal is coordinated in the TAOPP porphyrin center. 

The spectra of the silica hybrid materials are similar to the main important characteristics, as follows: each silica hybrid sample exhibits a broad band at 3450 cm^−1^ and 1610–1630 cm^−1^ due to O-H combined bending and stretching vibrations from adsorbed water on the surface of silica matrices [31]. All the silica hybrid materials exhibit a very intense and large band in the region between 1000 and 1250 cm^−1^, assigned to Si-O-Si vibrations. The pronounced shoulder at 1290 cm^−1^ present in each FT-IR spectrum of the silica hybrid materials is caused by Si-O-C_2_H_5_ and Si-C conjugated bonds.

In all silica samples, the fingerprint band of each porphyrin appears around 800 cm^−1^, due to C-H bonds in the pyrrole ring, covered by the intense and broad bands of Si-O-Si and Si-O-C [31]. The shoulder at 570 cm^−1^ in the FT-IR spectra of the hybrids containing platinum might reveal a possible generation of weak Pt-O-silica interaction [35].

#### 2.1.5. Atomic Force Microscopy (AFM) Analysis

**Figure 6:**
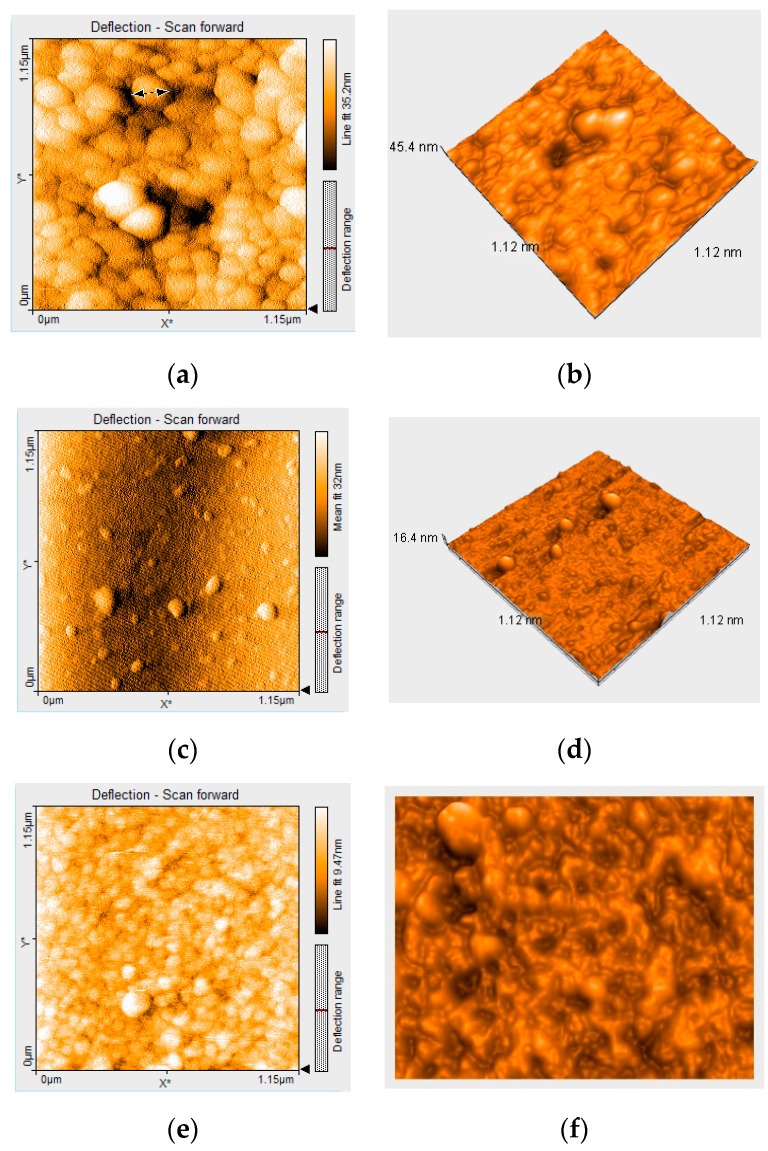
Atomic force microscopy (AFM) deflection and 3D image of: (**a**,**b**) PtTAOPP-silica hybrid material; (**c**,**d**) PtNPs-silica hybrid; (**e**,**f**) (TAOPP-PtNPs)-silica hybrid.

A head to tail arrangement of the initial spherical aggregates, equally generated by interactions between hydrophobic porphyrin molecules and by hydrogen bonds formed between porphyrin functionalities (capable for both hydrogen accepting and for hydrogen donating) and silica precursors, can be easily seen in the PtTAOPP-silica hybrid material (Figure 6a,b). Voids of large dimensions equally distributed make these hybrid materials a promising substrate for gas detection or even storage. The diameters of the spheres are in the range of 85–157 nm with small surface roughness (Sa) of 1.8 nm, a peak height value (Sp) of 9.8 nm and a valley depth value (Sv) = −11 nm. The 3D image reveals hemispheres from the interface. 

In the case of PtNPs-silica hybrid material, the 3D image (Figure 6c,d) clearly shows the isolated spheres from the surface. The aggregation is avoided due to absence of porphyrin from this material. The diameters of the spheres are smaller at 44–94 nm, while the surface roughness, Sa, is ten times higher, at 18 nm, and it has a peak height value, Sp, of 37 nm and a valley depth value, Sv = −57 nm.

With respect to the (TAOPP-PtNPs)-silica hybrid material (Figure 6e,f), the morphology is similar to that of PtTAOPP-silica hybrid material, probably because the PtNPs get accommodated in the core of the TAOPP porphyrin. The diameters of the spheres are in the range of 98–107 nm, while the surface roughness, Sa, is ten times higher at 12 nm, and it has a peak height value, Sp, of 24 nm and a valley depth value, Sv = −27 nm. Concave/convex shapes can be observed in the tridimensional analysis and the presence of PtNPs spheres on the surface of the material is diminished.

#### 2.1.6. High Resolution Transmission Electron Microscopy (HRTEM)

**Figure 7:**
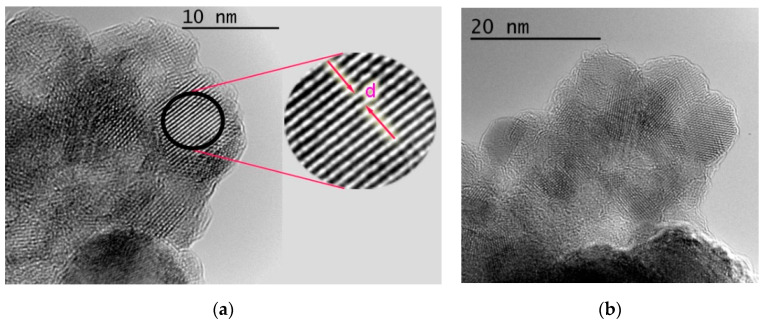
High resolution transmission electron microscopy (HRTEM) image for PtTAOPP-silica hybrid at different scales: (**a**) 10 nm and (**b**) 20 nm.

High resolution transmission electron microscopy (HRTEM) images of PtTAOPP-silica hybrid show amorphous and uniformly distributed lacey fractal structures [36] interrupted by crystalline zones with irregular arrangement. The marked distance of the straight fringes in the silica framework is around 0.29 nm (Figure 7a). Heterogeneities in the mesostructuraled ordering of the membranes are arising from non-uniform drying (Figure 7b) [37].

#### 2.1.7. Morphological and Textural Analyses

**Figure 8:**
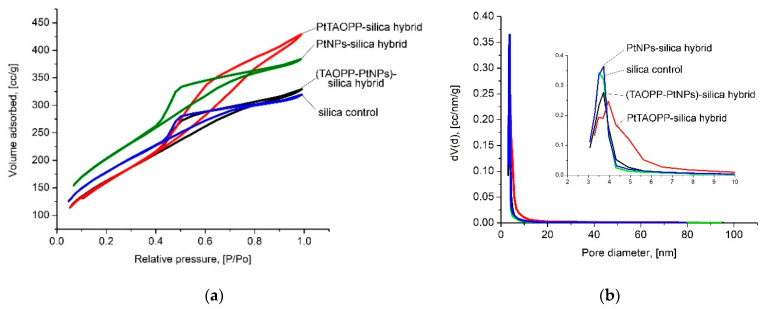
(**a**) N_2_ adsorption-desorption isotherms and (**b**) pore size distribution for all samples.

Morphological and textural analyses were determined by investigating nitrogen absorption/desorption isotherms, represented in Figure 8. The total pore volume was attained from the last point of the isotherm. 

From Figure 8, comparing the data with recent IUPAC recommendations [38], it can be observed that all isotherms are type IVa. The presence of the hysteresis shows that capillary condensation occurs. 

By analyzing the isotherms, it can be found that PtTAOPP-silica hybrid sample has the specific behavior of type H2b hysteresis that is a characteristic of ink bottle mesopores with large necks, having a small fraction of spherical pores, as confirmed by HR-TEM [36].

In the case of all other samples, we obtained a H4 hysteresis specific for ordered mesopores, accompanied in small extension by narrow slit-like micropores with irregular-shaped voids [39]. The most significant morphological characteristics, obtained from analyzing the isotherms, such as specific surface aria, pore size diameter, total pore volume and fractal dimensions, are shown in Table 2. Fractal dimensions were obtained using the Frenkel–Halsey–Hill (FHH) method [40].

Higher pore volumes were observed for the samples containing platinum, no matter whether PtNPs or Pt-porphyrin were used. The platinum-containing samples also had a fractal dimension with smaller values than those of the control silica sample. When the values of fractal dimension are close to 2, this indicates a smoother surface, which is a characteristic of the PtTAOPP-silica hybrid material.

The PtNPs-silica hybrid material was characterized by the highest surface area and micropore area but, in the case of total pore volume, the highest value was attained also by the PtTAOPP-silica hybrid material.

### 2.2. Silica Hybrid Materials Applied for CO_2_ Detection/Storage

**Figure 9:**
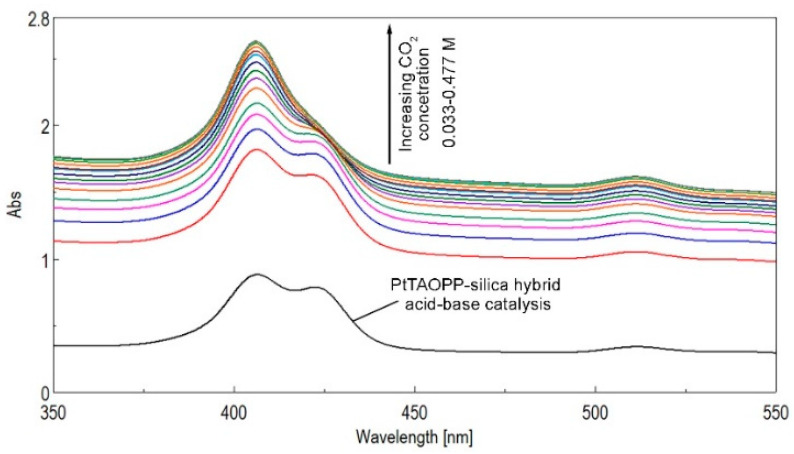
The effect of adding CO_2_ gas to PtTAOPP-silica hybrid, monitored by UV-vis spectroscopy.

In order to test the CO_2_ detection capacity of PtTAOPP-silica hybrid, the acid-catalyzed synthesis step was performed as in Section 3.3. For this particular case, the synthesis was stopped after adding only the amount of NH_3_ to prevent instant gelifiation and to reach the pH = 6 of the sol. The color of the sol changed from orange to light red. The testing solution was obtained by dissolving 4 mL of PtTAOPP-silica hybrid sol thus obtained in 50 mL of a mixed solution THF:water = 2:3 (V/V). The experiments were realized by introducing the testing solution in a three-neck flask with entries for CO_2_, exits for sampling and an outlet for the pressure equalizer. CO_2_ gas was introduced to the stirred testing solution with a rate of 100 mL/min, and UV-vis monitoring was realized by taking samples every five minutes (Figure 9).

By increasing the CO_2_ concentration, the intensity of the absorption bands of the PtTAOPP-silica hybrid material was continuously increasing, as shown in Figure 9, and the two initial Soret bands located at 410 and 450 nm merged into only one large band with the peak positioned at 410 nm. Because of the acid environment produced by adding CO_2_, porphyrin aggregates of both H-type and J-type were formed, causing the increase in intensity of the PtTAOPP-silica bands in the UV-vis spectra [28]. 

**Figure 10:**
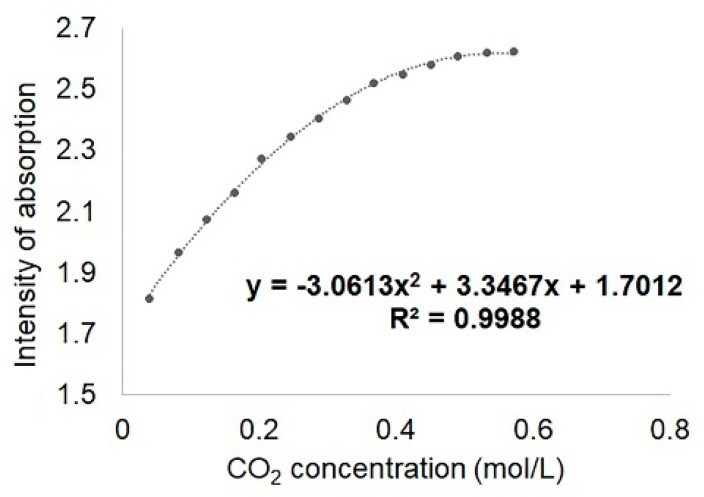
The dependence between the intensity of the Soret band measured at 410 nm and the CO_2_ concentration, for 95 min gas flow.

The dependence between the intensity of the Soret band measured at 410 nm and the CO_2_ concentration is given below in Figure 10, as a polynomial equation with a confidence coefficient of 99.88%.

**Figure 11:**
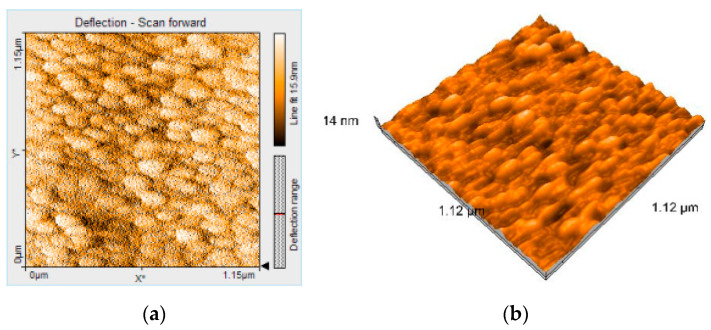
Atomic force microscopy 2D image (**a**) and 3D image (**b**) of PtTAOPP-silica hybrid material after CO_2_ detection.

The AFM image of PtTAOPP-silica hybrid after CO_2_ detection presented highly ordered ovoid-type structures, oriented in rows and with particle dimension of 98 nm (Figure 11a,b). This self-rearrangement and stratification of the material after contact with CO_2_ gas is the proof of significant interactions with amorphous PtTAOPP-silica hybrid surface. 

Similar tests have been performed with all the other obtained silica hybrid materials: PtNPs-silica hybrid, (TAOPP-PtNPs)-silica hybrid and silica control and the CO_2_ adsorption capacities are presented in the last four lines in Table 3. Table 3 also presents a comparison of the pore size and the CO_2_ adsorption capacities of different reported porphyrin- and silica materials and of the materials tested in this work.

As expected, from its morphological characteristics (Table 3) and from the presence of inkbottle mesopores with large necks, PtTAOPP-silica hybrid exhibited the second best performance of CO_2_ recovery, after that already reported [48] using Rh-metalloporphyrin metal-organic framework. In addition, when this material is used for CO_2_ detection, the range of detection is significantly extended to higher concentrations than reported by previous studies [8,46], as shown in Table 3. This is a promising result for further applications in CO_2_ detection for monitoring environmental conditions [8] or industrial processes. Anyway, minor changes regarding the morphology of the material were observed after the interaction with CO_2_. For instance, specific surface area was diminished to 446 ± 15 m^2^/g, pore size diameter was decreased to 3.68 nm, but the Frenkel–Halsey–Hill (FHH) fractal dimension remained almost the same (2.25). The PtTAOPP-silica hybrid material also preserved the shape of the pores. Taking this in consideration, we have to design an inexpensive disposable sensor. 

It seems that the presence of PtNPs in the hybrids, solely or accompanied by TAOPP, diminished the absorption capacity for CO_2_ gas by half, although the specific surface area and the dimension of pores of the PtNPs-silica hybrid leads us to expect another hierarchy.

Based on these results, we can presume that the process is not based only on physical adsorption but is accompanied by other physical–chemical interactions, depending on the dispersibility of the porphyrin into the silica matrices. In addition, due to the fact that no linear dependence was obtained to pass through the origin point (Figure 11), this is a proof for an intraparticle diffusion mechanism, and it is clear that intraparticle diffusion is not the sole factor controlling CO_2_ adsorption rate [49]. The temperature of 20 °C was chosen to favor CO_2_ adsorption, which is an exothermic reaction.

### 2.3. Testing of PtTAOPP-Silica Hybrid Material and of Silica Control for Methylene Blue Removal from Wastewaters

Based on large extent of previously reported results [4,26], another expected application of these novel hybrid materials was for the removal of harmful/toxic dyes from solutions. 

#### 2.3.1. Method Applied for the Adsorption of Methylene Blue (MB) from Wastewaters

In order to test the capacity of the hybrid materials to remove dyes from wastewaters, sets of test tubes were prepared as further described. 

A precisely weighted quantity of 0.0165 g from either the silica control or PtTAOPP-silica hybrid material was introduced into test tubes, then 2.5 mL of 0.1 M NaOH solution and 2.5 mL of different concentrations in the range 1 × 10^−3^–1 × 10^−6^ M methylene blue solved in water were added. To obtain a loading of 3.3 g/L, a quantity of 0.0165 g was weighed. The mixtures as prepared were stirred for 1 min and then left for 6 h to observe the removal of color. 

**Figure 12:**
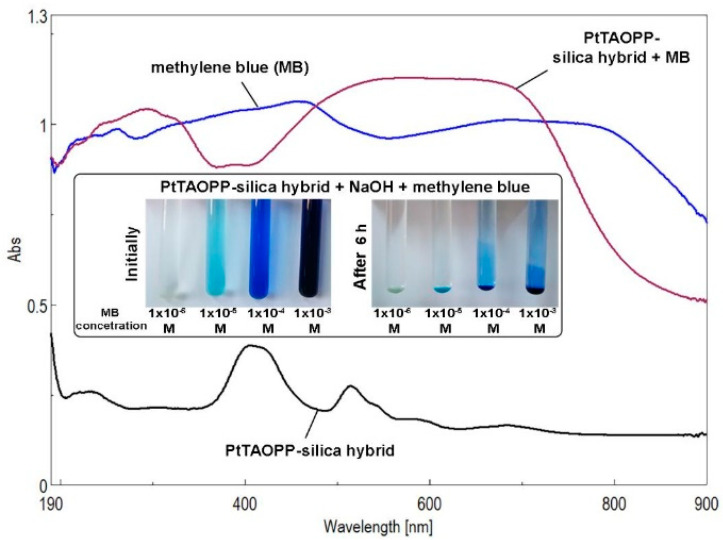
UV-vis spectra regarding the methylene blue (MB) recovery from wastewaters by solid PtTAOPP -silica hybrid sample, for a contact time of 6 h. Photographs demonstrating the removal of color before and after a period of 6 h as a function of the MB concentration.

Figure 12 shows the UV-vis spectra of the solid PtTAOPP-silica hybrid material before and after contact with the following MB concentrations: 1 × 10^−3^, 1 × 10^−4^, 1 × 10^−5^, and 1 × 10^−6^ M. An inset with the color removal of MB solution is displayed in the same image. The spectrum of PtTAOPP-silica hybrid presents low intensity peaks located at 406 and 510 nm, respectively, attributed to the PtTAOPP moiety. The spectrum of the PtTAOPP-silica hybrid after MB adsorption presents an intense and large plateau located between 450 and 700 nm covering the absorption intensities of both the PtTAOPP porphyrin and of MB. This is the proof of the interaction between the adsorbent PtTAOPP-silica hybrid and the MB dye. The high basicity (pH = 13) of the solution increases the number of negatively charged hydroxyl groups from silica matrix, facilitating the attraction between the dye and adsorbent surface. 

The significant color removal of the solutions in contact with PtTAOPP-silica hybrid material or silica control as well as the modified UV-vis spectrum of the solid samples after dye adsorption justified the investigations concerning the kinetics of the adsorption process. A comparison of the MB adsorption performances on silica control and PtTAOPP-silica hybrid, presented in Table S1 from Supplementary Material, shows that, regardless of the mass of adsorbent that was used, the adsorption capacity of PtTAOPP-silica hybrid is similar or 12–20% higher in the case of PtTAOPP-silica hybrid. In addition, at higher concentrations than 5 × 10^−5^ M MB, the PtTAOPP-silica hybrid is capable of adsorbing a higher amount of dye than the silica control, after 20 min contact.

#### 2.3.2. Results of the Kinetic Studies for the Adsorption Process of MB by Silica Control and by PtTAOPP-Silica Hybrid

The kinetic studies are presented in detail in Supplementary Material, as briefly described below. Time course measurements were performed in order to establish the discoloration of the dyes as a function of contact time, as represented in Figure S1. The variation in time of the amount of dye adsorbed using three different adsorbent loadings is presented in Figure S2. Table S1 presents the influence of adsorbent mass upon the adsorption capacity of silica control and PtTAOPP-silica hybrid. Our experimental results clearly show that the maximum of MB uptake was obtained for both investigated adsorbents at a loading of 0.83 g/L. The influence of the initial concentration of MB and contact time onto the removal of MB by the two adsorbent materials was presented in Figure S3, and Table S2. The equations used for establishing if pseudo-first order or pseudo-second order kinetic studies are fit to describe our results are shown in Figures S4 and S5. Figure S6 and Table S3 present the desorption capacities of the material in different media, by comparison to literature data. 

Table 4 summarizes the kinetic parameters for the adsorption of methylene blue by the silica control and by the PtTAOPP-silica hybrid at different loadings, for pseudo-first order kinetics and for second order kinetics. The deviations in the values of the pseudo first order rate constant k_1_ with PtTAOPP-silica hybrid loading show that the adsorption rate increases with the decrease in adsorbent loading. The calculated values of q_e_ in the case of pseudo-first order kinetics differ from the experimental results, and therefore the first-order kinetic model is not appropriate to explain the rate process. Differences in the values of the rate constant of pseudo-second order adsorption, *k_2_* parameter, for different adsorbent loadings for both investigated materials are observed. In addition, the adsorption rate *h* (mg × g^−1^ × min^−1^) increases with the decrease in adsorbent loadings, for both materials studied. It can be concluded that the pseudo-second order kinetic model fits better with the experimental data for both materials due to the fact that the calculated q_e_ values are closer to the experimental ones. This observation leads to the conclusion that the adsorption of MB on the investigated silica materials is accompanied by chemical interactions between adsorbent and adsorbate [50]. It can also be noticed that PtTAOPP-silica hybrid material is a better adsorbent for methylene blue than the silica control, probably due to the presence of the porphyrin moiety that facilitates chemical interactions with the dye molecule. 

The silica control and the PtTAOPP-silica hybrid do not perform as well as other studied adsorbent materials: neem leaves: 8.76–19.61 mg/g [51], corn husk: 9.34 mg/g [52] fly ash: 13.42 mg/g [53], but these materials are of natural origin, from different locations and their composition and capacity of adsorption may vary considerably with the method of treatment they are subjected to, before the adsorption tests. In our case, the adsorption tests were performed without exposure to light and without constant stirring, by simple contact of the interfaces, meaning that our method can be easily applied in any polluted environment. 

## 3. Materials and Methods

### 3.1. Reagents

Tetraethyl orthosilicate (TEOS) and ethanol absolute (EtOH) were provided by Fluka, tetrahydrofuran (THF) was purchased from Merck (Darmstadt, Germany) and all were *purrum analyticum* grade. TAOPP was obtained as previously reported [31,54,55]. Pt-TAPP was synthesized by following already optimized reported methods [55,56]. The PtNPs were obtained according to a literature procedure [57] using an aqueous solution of H_2_PtCl_6_ × 6H_2_O reduced with trisodium citrate, followed by reduction with NaBH_4_, until the color of the mixture changed from yellow to dark brown. The PtNPs nanoparticles had dimensions in the range of 44–94 nm.

### 3.2. Apparatus

UV-visible spectra were recorded on a JASCO V-650 apparatus in cells with 1 cm pathlength. FT-IR spectra were performed after preparing KBr pellets on a JASCO 430 FT-IR apparatus in the 400–4000 cm^−1^ range. For registering the emission spectra LS-55, PerkinElmer/UK equipment was used at a rate of 100 nm/min, setting the excitation slit at 10 nm and the emission slit at 4 nm, at room temperature in quartz cuvettes of 1 cm width for liquid samples and in a copper sample holder for solid samples.

Retsch Mixer Mill MM200 equipment with grinding jars performing radial oscillations in a horizontal position was used to obtain fine powder samples.

Morphological and textural analyses were determined after investigating the nitrogen isotherms at 77 K, on a QuantachromeNova 1200 apparatus. From the nitrogen adsorption isotherm curves, the specific surface area (SBET), average pore diameter (Dp) and total pore volume (Vp) were determined. The Frenkel–Halsey–Hill theory (FHH) and equations were used to calculate the surface roughness based on the van der Waals attraction between the solid and adsorbed film, which tends to replicate the gas–film interface [58]. Before analyzing in nitrogen at 77 K, the samples were degassed at 55 °C in vacuum for 6 h. In order to obtain the surface area, the BET (Brunauer–Emmett–Teller) method [31] was used, and pore size distribution was obtained by the BJH (Barrett, Joyner, and Halenda) method [58]. The micropore area was determined using the V-T method [59]. 

The atomic force microscopy (AFM) images of the hybrid porphyrin-silica materials deposited on silica plates were taken in the contact or tapping mode on the Nanosurf^®^ EasyScan 2 Advanced Research AFM, using a piezoelectric ceramic cantilever with a (450 mm × 50 mm × 2 mm) stiff. AFM data were quantitative on all three dimensions, with the dark colors and their light tones representing the lower and the higher features, respectively. HR-TEM analyses were investigated on TEM/STEM Titan G2 80-200 apparatus operating at 200 kV and visualized by Digital Micrograph v. 2.12.1579.0 software. The silica powders were highly dispersed in EtOH for 30 min and deposited on TEM copper grids (300 mesh) covered with carbon film.

### 3.3. In Situ Two Steps Acid/Base Catalyzed Sol-Gel Method for Obtaining Silica Hybrids

A solution was made from 10.42 g (0.05 mol) TEOS and 11.68 mL (9.22 g, 0.2 mol) EtOH and vigorously stirred. After 15 min, 0.082 mL (0.037 g, 0.001 mol) of a solution of 37% HCl was added by slow dropping, under vigorous stirring. The following molar ratios were used during the first acidic step: TEOS:EtOH:H_2_O:HCl = 1:4:6:0.02. After another 15 min, the second base-catalytic step was started by slowly adding a solution of NH_3_ 2.5% until the viscosity increased. This is the moment when the three different impregnated matrices were obtained, as follows:

1. For the obtaining of the PtTAOPP-silica hybrid, 10 mL of a solution of 2.1 × 10^−6^ M PtTAOPP in THF (molar ratio: TEOS:PtTAOPP = 25,000:1) was added slowly under stirring.
2. For the obtaining of the PtNPs-silica hybrid, 5 mL of 4.2 × 10^−6^ M Pt colloidal solution was added to respect the same molar ratio regarding TEOS (TEOS:PtNPs = 25,000:1).
3. For the obtaining of the (TAOPP-PtNPs)-silica hybrid, a mixture composed from 10 mL of a solution of 2.1 × 10^−6^ M PtTAOPP in THF and 5 mL of 4.2 × 10^−6^ M Pt colloidal solution was added, maintaining the same molar ratio: TEOS:PtNPs:TAOPP = 25,000:1:1.
4. The control silica sample was identically synthesized, without the addition of any porphyrin derivatives or PtNPs. A transparent gel was the resulting product.

In all three cases, the basic catalysis was controlled by slowly adding 2.5% NH_3_ solution until instant gelation occurred (3 mL for PtTAOPP-silica hybrid; 1.5 mL for PtNPs-silica hybrid and 4 mL for (TAOPP-PtNPs)-silica hybrid, respectively).

**Figure 13:**
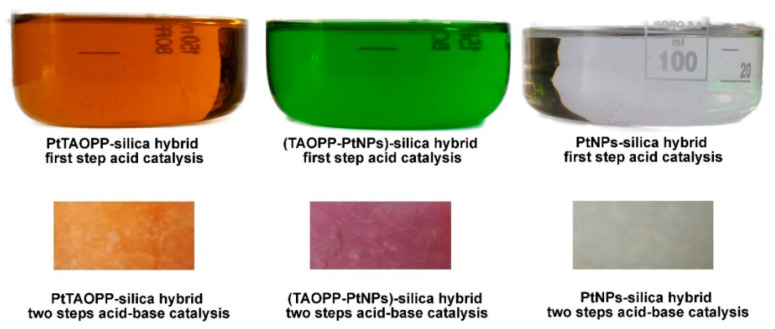
The colors of silica hybrid materials function of the nature of partner and the moment of acid first step or base –second step catalysis.

The final products were stable gels of different colors, as presented in Figure 13; orange for PtTAOPP-silica hybrid; dark-pink for (TAOPP-PtNPs)-silica hybrid and colorless for PtNPs-silica hybrid, respectively. After the wet gels were dried at 100 °C for 8 h, they were ground in a mill.

## 4. Conclusions

The main purpose of this work was to obtain novel porphyrin-silica hybrid materials with high chemical stability, large specific surface areas, tailored pore shapes and sizes and to use them for the detection/adsorption of carbon dioxide and for the removal of dyes from waste waters. The silica gels were prepared starting from TEOS using the sol-gel method performed in two step acid-base catalysis. We incorporated PtTAOPP and PtNPs alone and accompanied by TAOPP in the silica gels. A comparison between the newly obtained hybrids shows that by the incorporation of PtTAOPP into the silica matrix, the optical and morphological properties of the hybrid are improved, although the PtTAOPP content is 25,000 times lower than that of TEOS. 

The capacity of these new hybrid materials to adsorb/detect carbon dioxide was investigated. The PtTAOPP-silica hybrid material, having suitable morphologic characteristics (Table 2), is able to adsorb the highest amount of CO_2_, that is, 0.025 mol CO_2_/g, representing the second best performance of CO_2_ recovery, after that already reported [48]. The other investigated materials also have remarkable but lower adsorption capacities, namely: silica control 0.021 mol/g, (TAOPP-PtNPs)-silica hybrid 0.016 mol/g and PtNPs-silica hybrid 0.011 mol/g, respectively. In addition, when PtTAOPP-silica hybrid material is used for CO_2_ detection, the range of detection is significantly extended to higher concentrations than reported by previous studies [8,46], as shown in Table 3. This is a promising result for further applications in CO_2_ detection for monitoring environmental conditions [8] or industrial processes. 

Methylene blue adsorption tests were performed on the silica control and on the PtTAOPP-silica hybrid and showed adsorption capacities similar to those mentioned in the literature, depending on MB concentrations and the mass of adsorbent materials. The kinetic studies performed in the time course measurements showed that a second order kinetic model is suitable to describe the adsorption process for both studied silica materials, proving that both physical and chemical adsorption processes are simultaneously involved. 

## Figures and Tables

**Table 1:** The materials used to obtain silica hybrids and their abbreviations.

Silica Base	Immobilized Component	Hybrid Abbreviation
Tetraethyl orthosilicate (TEOS)	Pt(II)-5,10,15,20-tetra-(4-allyloxy-phenyl)-porphyrin (PtTAOPP)	PtTAOPP-silica hybrid
TEOS	5,10,15,20-tetra-(4-allyloxy-phenyl)-porphyrin (TAOPP) and Pt nanoparticles (PtNPs)	(TAOPP-PtNPs)-silica hybrid
TEOS	Platinum nanoparticles (PtNPs)	PtNPs-silica hybrid
TEOS	-	Silica control

**Table 2:** The most significant morphological characteristics obtained from N_2_ adsorption/desorption isotherms.

Name	Specific Surface Area [m^2^/g]	Pore Size Diameter [nm]	Total Pore Volume [cc/g]	Frenkel-Halsey-Hill (FHH) Fractal Dimension
PtTAOPP-silica hybrid	593 ± 15	4.12	0.66	2.20
PtNPs-silica hybrid	739 ± 19	3.73	0.59	2.45
(TAOPP-PtNPs)-silica hybrid	592 ± 15	3.71	0.51	2.41
Control silica	650 ± 17	3.51	0.49	2.50

**Table 3:** Comparative CO_2_ adsorption capacities (20 °C) or detection ranges provided by silica- or porphyrin-based materials, reported in recent literature and in this work.

Silica- or Porphyrin- Based Materials	Pore Size Diameter (nm)	CO_2_ Adsorption (mmol/g)	Range of Detection (mM)	Ref.
Mesoporous silica obtained from TEOS and a triblock copolymer poly(ethylene oxide)-poly(propylene oxide)-poly(ethylene oxide) impregnated with 50 wt % polyethyleneimine	5.3	3.068	-	[41]
Circulating Fluidized Bed method with mesoporous silica having BET surface area of 250 m^2^/g and pore volumes of 1.7 c^3^/g impregnated with 40 wt % polyethyleneimine	20	1.65	-	[16]
TEOS-based mesoporous silica impregnated with 70 wt % polyethyleneimine	-	2.045	-	[17]
Silica gel from sodium silicate activated with 2-[2-(3-Trimethoxysilylpropylamino)-ethylamino] ethylamine	6	4.773	-	[42]
Mesoporous silica gel from sodium silicate modified with propilamine	67	2.3	-	[43]
Silica aerogels starting from TEOS, modified with (3-aminopropyl) triethoxysilane	1.27	2.87 ± 0.05	-	[44]
TEOS-based silica matrix impregnated with 5,10,15,20-tetratolyl-21H,23H-porphyrin	1.37–2.60	3.089	1.21–4.5	[18]
TEOS based silica material impregnated with 5-(4-carboxyphenyl)-5,10,15-tris(4-phenoxyphenyl)-porphyrin and Fe_3_O_4_ magnetic nanoparticles	-	-	3 × 10^−2^–0.20	[45]
5-(4-pyridyl)-10,15,20-tris(3,4-dimethoxyphenyl)-porphyrin	200	-	49–306	[8]
Colorimetric sensor film based on phosphorescent Pt(II) meso-Tetra(pentafluorophenyl)porphine, poly(isobutyl methacrylate), α-naphtholphthalein and cetyltrimethylammonium hydroxide	-	-	1.51–30.3 (2 to 40% in ethanol and olive oil)	[46]
Porous crystalline framework based on 5, 10, 15, 20-tetra-(2-quinolyl)-21H, 23H-porphine (meso-tetra-2-quinolyl-porphyrin	0.46	0.18	-	[47]
Metal-organic framework built from Rh- tetrakis(4-carboxyphenyl)porphyrin and ZrCl_4_	1.85	44.2	-	[48]
PtTAOPP-silica hybrid material	4.12	25 ± 0.05	40–570	This work
PtNPs-silica hybrid material	3.73	11 ± 0.06	-	This work
(TAOPP-PtNPs)-silica hybrid material	3.71	16.3 ± 0.06	-	This work
TEOS-based silica control	3.51	21.6 ± 0.07	-	This work

**Table 4:** Kinetic parameters for the adsorption of methylene blue by silica control and by PtTAOPP-silica hybrid at different loadings.

Equations	Parameters	Silica Control			PtTAOPP-Silica Hybrid		
		0.83 g/L	1.66 g/L	3.33 g/L	0.83 g/L	1.66 g/L	3.33 g/L
**Pseudo-first order**	q_e_ exp. [mg/g]	6.921	3.611	4.803	7.261	3.656	4.803
	q_e_ calc. [mg/g]	-	-	-	15.26 ± 1.0	9.603 ± 0.9	14.429 ± 1.2
	*k*_1_[min^−1^]	-	-	-	0.0838	0.048	0.022
**Pseudo-second order**	q_e_ calc. [mg/g]	7.001 ± 1.1	3.868 ± 0.3	5.04 ± 0.5	7.31 ± 0.5	3.709 ± 0.1	4.853 ± 0.2
	*k*_2_[g × mg^−1^ × min^−1^]	0.18	0.08	0.03	0.19	0.157	0.025
	*h* [mg × g^−1^ × min^−1^]	8.622	1.043	0.69	10.016	2.099	0.576

## Supplementary Materials

Supplementary materials can be found at https://www.mdpi.com/1422-0067/21/12/4262/s1.
