# Supplementary material for: Hybrid Materials Based on Silica Matrices Impregnated with Pt-Porphyrin or PtNPs Destined for CO2 Gas Detection or for Wastewaters Color Removal
Source: Int J Mol Sci. 2020 Jun 15;21(12):4262. doi: 10.3390/ijms21124262 (PMC7352184; doi:10.3390/ijms21124262)
Supplement: Supplementary file 1 [file ijms-21-04262-s001.pdf]

Hybrid materials based on silica matrices impregnated with Pt-porphyrin or PtNPs destined for CO_2 gas detection or for wastewaters color removal

Diana Anghel [1], Anca Lascu [1]*, Camelia Epuran [1], Ion Fratilescu [1], Catalin Ianasi [1], Mihaela Birdeanu [2] and Eugenia Fagadar-Cosma [1]*

[1] Institute of Chemistry "CoriolanDragulescu" of Romanian Academy, M. Viteazul Ave, No. 24,300223-Timisoara, Romania danghel@acad-icht.tm.edu.ro (D.A.); alascu@acad-icht.tm.edu.ro (A.L.); ecamelia@acad-icht.tm.edu.ro (C.E.); ionfratilescu@acad-icht.tm.edu.ro (I.F.); ianasic@acad-icht.tm.edu.ro (C.I.); efagadarcosma@acad-icht.tm.edu.ro (E.F.-C.);

[2] National Institute for Research and Development in Electrochemistry and Condensed Matter, P. Andronescu Street 1, 300224- Timisoara, Romania; mihaelabirdeanu@gmail.com (M.B.)

* Correspondence: ancalascu@yahoo.com or alascu@acad-icht.tm.edu.ro (A.L.); and efagadar@yahoo.com or efagadarcosma@acad-icht.tm.edu.ro (E. F.-C.); Tel.: +40-256-491-818

Kinetic studies regarding the adsorption of methylene blue (MB) by the silica control and PtTAOPP-silica hybrid

- Time course measurements

In order to establish decoloration of the dyes as a function of contact time, the samples were prepared in quarz cuvettes by adding 3 mL 0.05 M NaOH solution containing 0.5 x 10^{-4} M MB over 0.01 g adsorbent (representing a loading of 3.33 g/L). The pH was 13. The mixture was stirred on vortex for 5 seconds and the measurements always take place after 15 seconds from the contact of the dye solution with the adsorbent. Figure S1 presents the time course measurement determination.

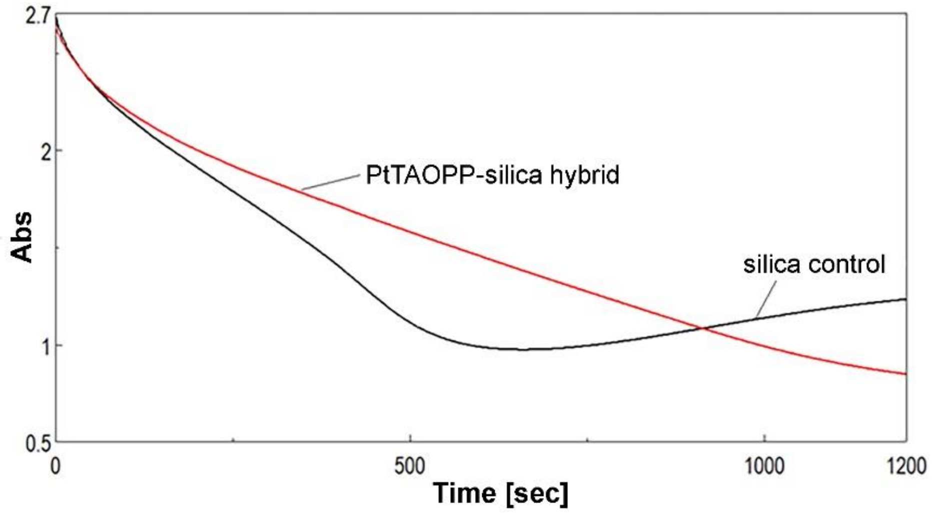

Figure S1. Time course measurements of the intensities of the samples containing methylene blue, in silica control and in PtTAOPP-silica hybrid adsorbent materials, at 664 nm.

During a contact time of 20 minutes, the silica control adsorbed 2.837 mg MB/g and the PtTAOPP-silica hybrid material 3.452 mg MB/g, respectively. This significant difference is also noticed from the allure of the time course measurement spectra, where it can be observed that a slight desorption

phenomenon takes place in the case of silica control after 656 seconds measurement, whereas the PtTAOPP-silica hybrid does not present such trend and is able to continue the adsorption process.

- The effect of silica adsorbent materials loading upon the adsorption of methylene blue

Three different silica adsorbent materials loadings: 0.83 g/L, 1.66 g/L and 3.33 g/L were used to investigate the influence of the adsorbent weight quantity upon the adsorption of MB having a fixed initial concentration of 5 x 10^{-5} M (16 mg/L). In each case, time course measurements of the intensity of absorption of methylene blue were performed at the wavelength of 664 nm, for 1200 seconds. Figure S2 (a and b) shows the variation in time of the amount of MB dye adsorbed for the three adsorbent loadings investigated, for silica control (a) and for PtTAOPP-silica hybrid (b).

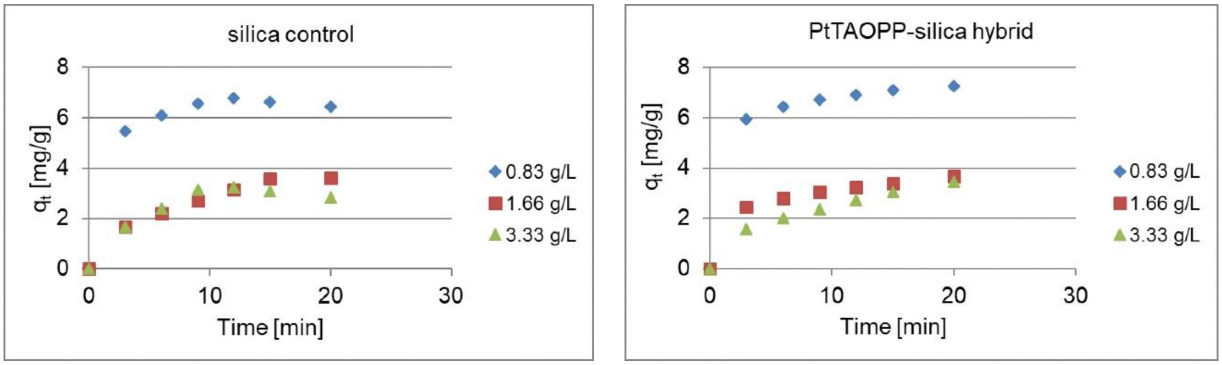

(**a**) (**b**)

Figure S2. The variation in time of the amount of dye adsorbed by the three adsorbent loadings investigated (**a**) silica control (**b**) PtTAOPP-silica hybrid

The equation (1) is used to calculate the amount of dye adsorbed by the investigated silica materials [1]:

$$q_e = \frac{C_0 - C_e}{m} \text{ (mg/g)} \tag{1}$$

where: q_e represents the amount of adsorbed dye (mg/g); C_o represents the initial concentration of dye in solution (mg/L); C_e represents the equilibrium concentration of dye (mg/L), that could be calculated by means of Lambert-Beer law and m represents the mass of sorbent (g/L). The percentage removal of dye at various times is calculated according to the equation (2)

$$\eta = \frac{C_o - C_t}{C_0} x100 \text{ (\%)} \tag{2}$$

From Figure S1 corroborated with Table S1 it can be observed that the PtTAOPP-silica hybrid can act as a better sorbent for MB than silica control during the time interval of 1200 seconds, at all the investigated loadings. In addition, the process of adsorbtion is continuing its ascendent alure after 1200 seconds in case of using as adsorption material the PtTAOPP-silica hybrid.

Table S1. Influence of adsorbent mass upon the adsorption capacity of silica control and PtTAOPP-silica hybrid, MB having a fixed initial concentration of 5 x 10^{-5} M (16 mg/L).

Adsorbent	Mass of adsorbent [g/L]	Adsorption capacity $q_{20\ min}$ [mg/g]	Yield η [%]
Silica control	0.83	6.453±0.2	33.48
PtTAOPP-silica hybrid		7.261±0.2	37.67
Silica control	1.66	3.611±0.03	37.47
PtTAOPP-silica hybrid		3.656±0.6	37.94
Silica control	3.33	2.837±0.2	59.07
PtTAOPP-silica hybrid		3.452±0.4	71.86

In theory [2], the increase in removal percentage with the increase in adsorbent mass is expected, due to the increase in the number of sites available for adsorption. Our experimental results clearly show that the maximum of MB uptake was obtained for both investigated adsorbents at a quantity of 0.83 g/L. For this reason, this loading was further used for testing the effect of dye concentration and contact time upon the adsorption.

- The effect of varying of the initial MB concentration and contact time

The plots between the amount of adsorbed dye as a function of time, when using different MB concentrations (16 mg/L; 9.6 mg/L; 4.8 mg/L; 4.5 mg/L) are presented in Figure S3. From Figure S3 a remarkable and continuous adsorbtion of MB at its highest concentration of 16 mg/L can be noticed in case of using PtTAOPP-silica hybrid as adsorbent material. When using silica control, only the concentration of 12.4 mg/L shows an ascendent trend.

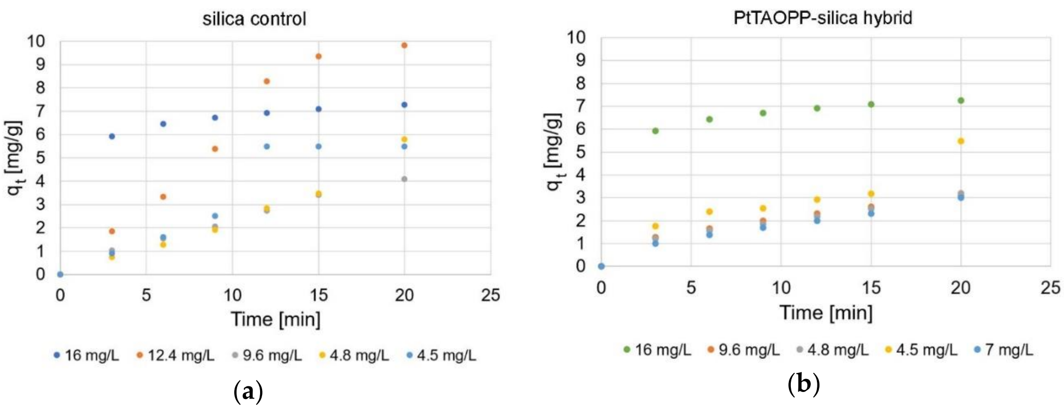

Figure S3. The influence of the initial concentration of dye and contact time onto the methylene blue removal (pH=13, 298K, 0.83 g/L adsorbent loading) for (**a**) silica control and (**b**) PtTAOPP-silica hybrid

From the data presented in Table S2, the influence of initial MB concentration on the adsorption capacities of silica control and PtTAOPP-silica hybrid for 0.83 g/L adsorbent loading, it can be observed that the percentage of dye removal decreases with the increase of the initial dye concentration, probably because of the rapid saturation of the binding sites of the adsorbent [1]. When using lower MB concentrations the silica control has a better adsorption capacity than PtTAOPP-silica hybrid, but at higher MB concentrations the PtTAOPP-silica hybrid is capable to adsorb a higher amount of dye (7.261 mg/g) than silica control (6.453 mg/g), after 20 minutes contact.

Table S2. Influence of initial MB concentration on the adsorption capacities of silica control and PtTAOPP-silica hybrid for 0.83 g/L adsorbent loading

Adsorbent	Initial MB concentration [mg/L]	Adsorption capacity $q_{20\ min}$ [mg/g]	Yield η [%]
Silica control	4.7	5.477±0.5	100
PtTAOPP-silica hybrid		5.477±0.4	100
Silica control	4.8	5.785±0.5	100
PtTAOPP-silica hybrid		3.192±0.4	55.21
Silica control	9.6	4.074±0.6	34.96
PtTAOPP-silica hybrid		3.094±0.4	27.54
Silica control	16	6.453±0.3	33.48
PtTAOPP-silica hybrid		7.261±0.2	37.67

- Adsorption kinetic study

Kinetic studies provide information concerning the mechanism of dye adsorption. According to [3] the pseudo-first-order kinetic of adsorption is represented by equation (3):

$$\frac{dq_t}{dt} = k_t(q_e - q_t) \tag{3}$$

where q_t is the amount of dye adsorbed at time t (mg/g); q_e is the adsorption capacity at equilibrium (mg/g); k_t is the pseudo first order rate constant (min^{-1}); t is the contact time (min). The integration of this equation with initial conditions (q_t = 0 at t = 0) leads to the following equation (4):

$$\log(q_e - q_t) = \log q_e - \frac{k_1}{2.303}t \tag{4}$$

The value for k_1 rate constant is calculated from the linear plots of log(q_e-q_t) versus t -Lagergren plots, as the slope of the plots [4].

The plots of log(q_e - q_t) vs. t (Figure S4 a,b) for the two adsorbent materials, silica control and PtTAOPP-silica hybrid respectively, at different loadings, show that the adsorption on silica control does not follow a first-order kinetic, but the straight-line plots for the case of PtTAOPP-silica hybrid indicate the validity of Lagergren equation. The calculated values of q_e differ from the experimental results, therefore the first-order kinetic model is not appropriate to explain the rate process.

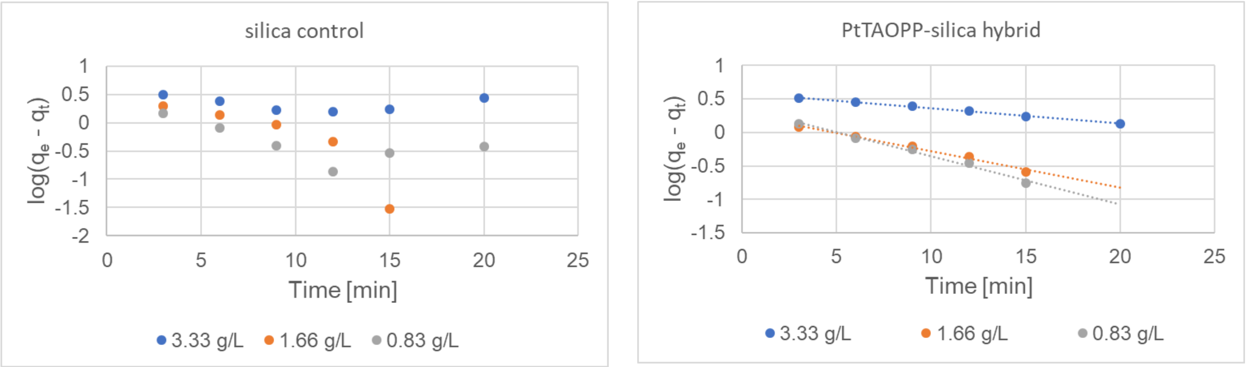

(a) (b)

Figure S4. Lagergren plots for the adsorption of Methylene blue on (a) silica control and (b) PtTAOPP-silica hybrid, at different loadings, at 25 °C

As a consequence, the pseudo-second order adsorption kinetic model was applied for both adsorbent materials and the rate constant of pseudo-second order adsorption was calculated from equation (5), representing the integrated pseudo-second order adsorption kinetic rate to initial conditions $q_t = 0$ at $t = 0$ [4]:

$$\frac{1}{q_e - q_t} = \frac{1}{q_e} + k_2 t \tag{5}$$

where k_2 is the rate constant of pseudo-second order adsorption (g x min^{-1} x mg^{-1}).

The rate constant for the second-order kinetic model, k_2 can be also calculated as the intercept of the linear plot t/q_t *vs* t, as can be seen in Figure S5 [3].

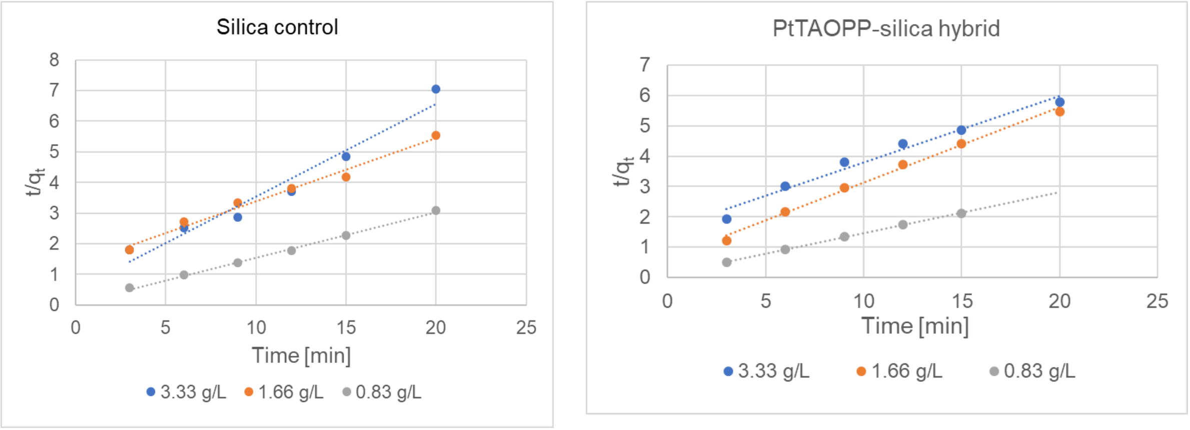

(**a**) (**b**)

Figure S5. Linear plots t/q_t *vs* t for (**a**) various loadings of silica control and (**b**) various loads of PtTAOPP-silica hybrid

The initial adsorption rate constant, *h* (mg x g^{-1} x min^{-1}) as $q_t/t \rightarrow 0$ at t = 0 can be defined as equation (6) [4]:

$$h = k_2 q_e^2 \tag{6}$$

The calculated values of q_e in the case of pseudo-first order kinetics differ from the experimental results therefore the first-order kinetic model is not appropriate to explain the rate process. The calculated q_e values for pseudo-second order kinetic model fit better with the obtained experimental data for both materials. This observation leads to the conclusion that the adsorption of MB on the investigated silica materials is accompanied by chemical interactions between adsorbent and adsorbate [7]. It can also be noticed that PtTAOPP-silica hybrid material is a better adsorbent for methylene blue than silica control, probably due to the presence of the porphyrin moiety that facilitates chemical interactions with the dye molecule.

Desorption studies of MB from PtTAOPP-silica hybrid

The desorption studies were performed as follows: portions of 0.0046 g PtTAOPP-silica hybrid after methylene blue adsorption were centrifuged, filtered and dried (6 h at 90 °C). These were further exposed to 2.5 mL eluent solutions: water, acetone, hydrochloric acid (0.5N), sodium hydroxide solution (0.5N), tetrahydrofurane and ethanol. The mixtures were stirred for two hours, then centrifuged and the UV-vis

spectra of the supernatant solutions were recorded. The amount of dye desorbed $q_{e,desorption}$ (mg/g) was calculated according to equation (7):

$$q_{e,desorption} = \frac{VxC_f}{M} \qquad (7)$$

where V-eluent solution volume (L), C_f – the dye concentration in the desorbing solution (mg/L), M-the saturated adsorbent weight (g). The desorption efficiency was calculated according to equation (8):

$$D\ \% = \frac{q_{e,desorption}}{q_{e,adsorption}}\ x\ 100 \qquad (8)$$

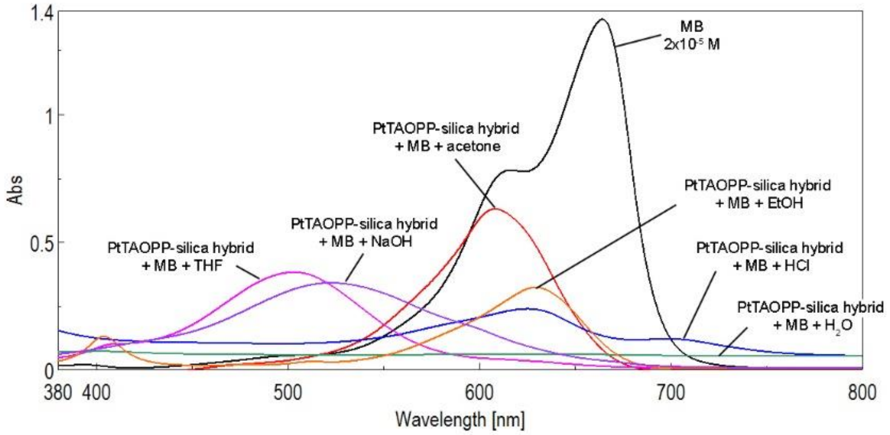

Figure S6. UV-vis spectra of the MB solutions after desorption experiments

Tabel S3. Desorption efficiency of MB from PtTAOPP-silica hybrid by using different types of solvents. Comparison of the desorption efficiency with literature data.

Material	Solvents used	Desorption efficiency [%]	References
PtTAOPP-silica hybrid	Water	2.21±0.1	This work
	Hydrochloric acid 0.5 M	8.54±0.3	This work
	Ethanol	11.66±0.5	This work
	Sodium hydroxide 0.5 M	12.27±0.6	This work
	Acetone	22.74±0.9	This work
	Tetrahydrofuran	27.48±1.2	This work
Brown macroalga	Water	2.36 ± 0.12	[5]
	Acetone	27.11 ± 1.41	
	Sodium hydroxide 0.5 M	0.00	
	Ethanol	21.92 ± 1.23	
Attapulgite/bentonite	Hydrochloric acid 0.1 M	1.94	[6]

We can conclude that the desorption efficiency of this material is slightly higher (27.48 %) than the best result reported in the literature (27.11 %).

References

1. Kannan, N.; Sundaram, M. M. Kinetics and mechanism of removal of methylene blue by adsorption on various carbons—a comparative study. *Dyes Pigm.* **2001**, *51(1)*, 25–40. doi: 10.1016/s0143-7208(01)00056-0

2. Barka, N.; Abdennouri, M.; Makhfouk, M.E. Removal of Methylene Blue and Eriochrome Black T from aqueous solutions by biosorption on Scolymus hispanicus L.: Kinetics, equilibrium and thermodynamics. *J. Taiwan Inst. Chem. E*. **2011**, *42(2)*, 320–326. doi: 10.1016/j.jtice.2010.07.004
3. Kaur, S.; Rani, S.; Mahajan, R. K. Adsorption Kinetics for the Removal of Hazardous Dye Congo Red by Biowaste Materials as Adsorbents. *Journal of Chemistry* **2013**, 1–12. doi: 10.1155/2013/628582
4. Lyubchik, S.; Lygina, E.; Lyubchyk, A.; Lyubchyk, S.; Loureiro J.M.; Fonseca I.M.; Ribeiro A.B.; Pinto, M.M.; Sa Figueiredo A.M. The Kinetic Parameters Evaluation for the Adsorption Processes at "Liquid–Solid" Interface. In: Electrokinetics Across Disciplines and Continents New Strategies for Sustainable Development, Ribeiro, Alexandra B., Mateus, Eduardo P., Couto, Nazaré, Eds. Springer, Cham, ISBN 978-3-319-20179-5. **2016**, pp. 81-109
5. Daneshvar, E.; Vazirzadeh, A.; Niazi, A.; Kousha, M.; Naushad, M.; Bhatnagar. A. Desorption of Methylene blue dye from brown macroalga: Effects of operating parameters, isotherm study and kinetic modeling. *J. Clean. Prod*. **2017.** *152,* 443-453. doi: 10.1016/j.jclepro.2017.03.119
6. Liu, Y.; Kang, Y.; Mu, B.; Wang, A. Attapulgite/bentonite interactions for methylene blue adsorption characteristics from aqueous solution. *Chem. Eng. J.* **2014,** *237*, 403–410. doi: 10.1016/j.cej.2013.10.048
7. Ngakou, S.C.; Anagho, G.S.; Ngomo, H.M. Non-linear Regression Analysis for the Adsorption Kinetics and Equilibrium Isotherm of Phenacetin onto Activated Carbons, *Curr. J. Appl. Sci. Technol*. **2019**, *36(4)*, 1-18. doi: 10.9734/cjast/2019/v36i430246
